# Multiplexed Nanopore-Based Nucleic Acid Sensing and Bacterial Identification Using DNA Dumbbell Nanoswitches

**DOI:** 10.1021/jacs.3c01649

**Published:** 2023-05-23

**Authors:** Jinbo Zhu, Ran Tivony, Filip Bošković, Joana Pereira-Dias, Sarah E. Sandler, Stephen Baker, Ulrich F. Keyser

**Affiliations:** † Cavendish Laboratory, University of Cambridge, JJ Thompson Avenue, Cambridge CB3 0HE, U.K.; ‡ Cambridge Institute of Therapeutic Immunology & Infectious Disease (CITIID), Jeffery Cheah Biomedical Centre, Cambridge Biomedical Campus, University of Cambridge, Cambridge CB2 0AW, U.K.; § School of Biomedical Engineering, Faculty of Medicine, Dalian University of Technology, No. 2, Linggong Road, Dalian 116024, China

## Abstract

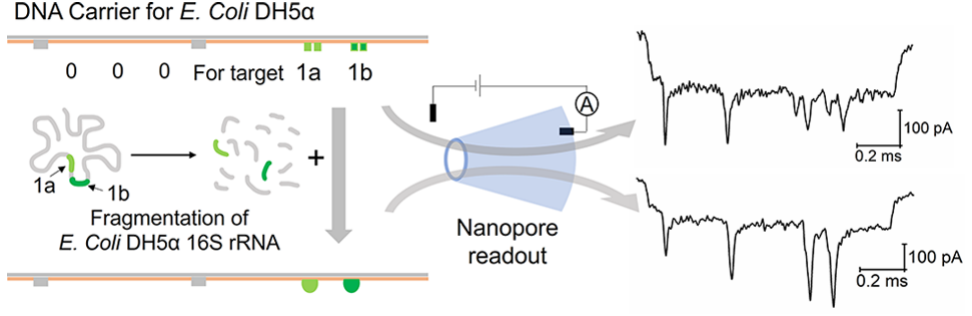

Multiplexed nucleic acid sensing methods with high specificity are vital for clinical diagnostics and infectious disease control, especially in the postpandemic era. Nanopore sensing techniques have developed in the past two decades, offering versatile tools for biosensing while enabling highly sensitive analyte measurements at the single-molecule level. Here, we establish a nanopore sensor based on DNA dumbbell nanoswitches for multiplexed nucleic acid detection and bacterial identification. The DNA nanotechnology-based sensor switches from an “open” into a “closed” state when a target strand hybridizes to two sequence-specific sensing overhangs. The loop in the DNA pulls two groups of dumbbells together. The change in topology results in an easily recognized peak in the current trace. Simultaneous detection of four different sequences was achieved by assembling four DNA dumbbell nanoswitches on one carrier. The high specificity of the dumbbell nanoswitch was verified by distinguishing single base variants in DNA and RNA targets using four barcoded carriers in multiplexed measurements. By combining multiple dumbbell nanoswitches with barcoded DNA carriers, we identified different bacterial species even with high sequence similarity by detecting strain specific 16S ribosomal RNA (rRNA) fragments.

## Introduction

Multiplexed detection of nucleic acids is vital for clinical diagnostics and genotyping, and has been made more relevant as a result of the recent COVID-19 pandemic.^1^ The analysis of genetic material can provide valuable information for identifying the pathogen type and providing targeted treatment.^2−4^ Due to the high similarity between the different variants of the same pathogenic species, high specificity is required for bacterial subtyping techniques.^5^ Besides the widely used polymerase chain reaction (PCR), loop-mediated isothermal amplification (LAMP), and DNA microarray assays,^6^ numerous novel nucleic acid detection methods have been developed using nanomaterials^7^ and enzymes. Examples include clustered regularly interspaced short palindromic repeats (CRISPR)-associated nucleases.^8^ However, these approaches often need specific enzymes, chemical modifications, fluorescent dyes, or time-consuming protocols. Thus, PCR-free and enzyme-free detection methods were proposed, such as DNA nanoswitches.^9−13^ The DNA nanoswitch is based on the conformational change of a DNA nanostructure that usually occurs in the presence of target biomolecules or ions. DNA nanoswitches have been monitored by various detection approaches, such as fluorescence,^14^ electrochemical sensing,^15^ single-molecule force spectroscopy,^16^ and atomic force microscopy (AFM).^17^

Recently, a gel electrophoresis-based label-free and amplification-free DNA nanoswitch for nucleic acid sensing with high specificity and sensitivity was developed.^18−20^ Upon binding a short single-stranded (ss) DNA or RNA, a loop forms on a long double-stranded (ds) DNA to indicate the presence of the target sequence. This sensing strategy has been applied in multiple fields including virus detection,^21^ RNA purification,^22^ and data storage.^23,24^ However, although the established gel-based topology readout method enables testing of the DNA nanoswitch, it is challenging to distinguish loops of the same size at different positions on the long strands in a gel. As a single-molecule technique, a nanopore readout could offer new advantages in multiplexing, material use, and speed of sensing.^25−30^ For instance, nanopore-based sensors have been successfully employed to detect topology changes caused by a single DNA nanoswitch^31^ and base pairing,^32^ as well as to determine the positions of various DNA nanostructures along a linearized M13 DNA strand (henceforth, a DNA carrier), by monitoring their time-dependent appearance during translocation.^33,34^ Furthermore, encoding each DNA carrier with a unique barcode for identification,^35^ e.g. by incorporating a logical arrangement of DNA dumbbell hairpins in a defined section on the carrier, allows the detection of multiple carriers using the same nanopore; therefore, the simultaneous detection of numerous target sequences can be achieved. However, in previous multiplexed nanopore sensing assays or data storage readout systems,^33,36−39^ signals were identified by the presence or absence of a current peak at a specific position during carrier translocation. Thus, missing peaks are prone to lead to both false positive and negative results partly caused by velocity fluctuations during DNA translocation.^40,41^ A more dynamic DNA nanostructure design that monitors changes in the structure should allow for multiplexed sensing with lower false positive rates.

**Figure 1:**
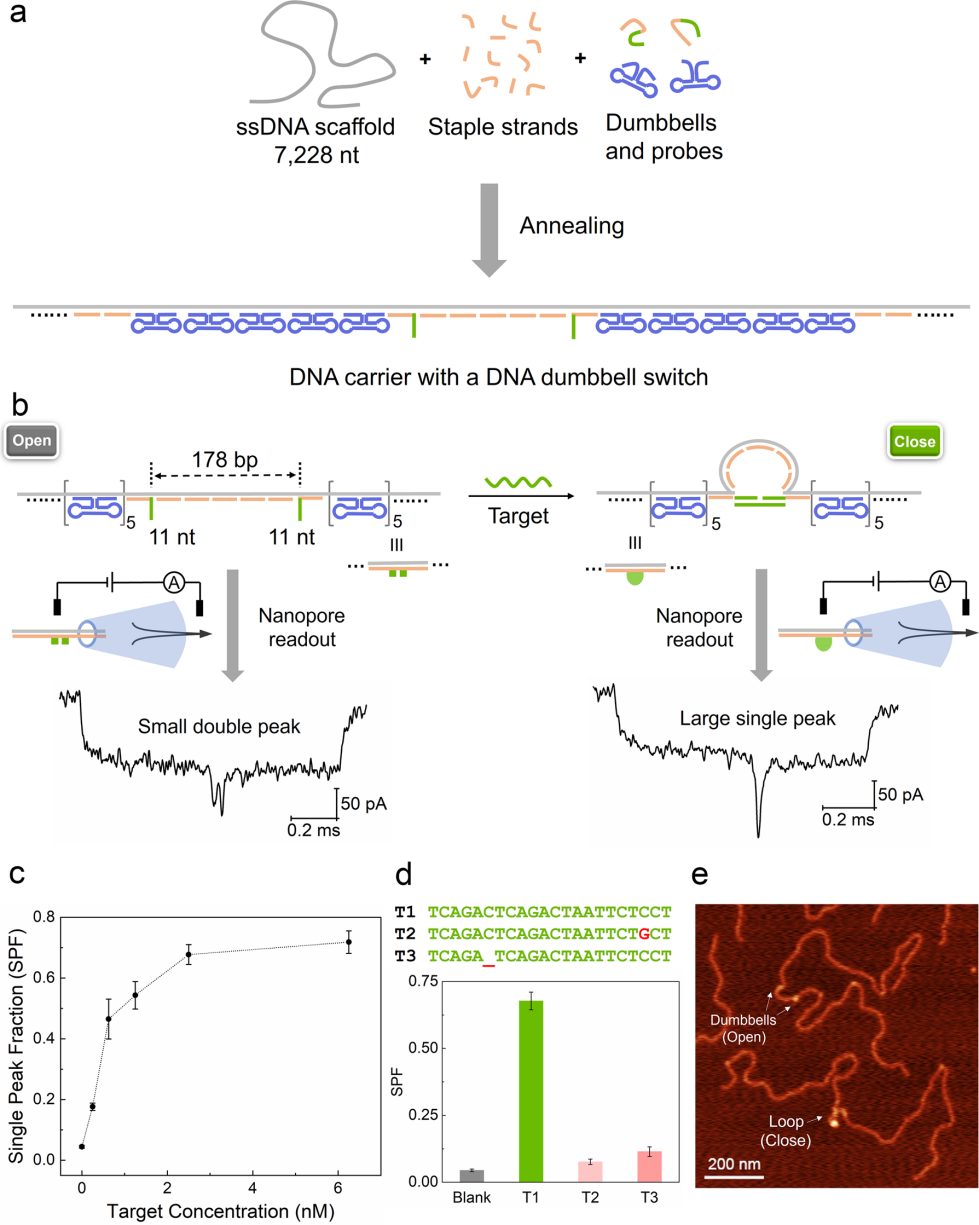
Detection of DNA target using DNA dumbbell nanoswitch-nanopore sensor. (a) Schematic of the self-assembly of DNA carrier with probes (green) and two groups of DNA dumbbells (blue). (b) Topological change of DNA dumbbell nanoswitch in the presence of a DNA target (strand T1) from “open” state to “close” state and the readout by nanopores with two example events. (c) Dependence of single peak fraction (SPF) on target concentrations. 0.125 nM DNA carrier is used for all measurements. SPF represents the percentage of “close” state in the two states of switch. (d) Detection of the single base substitution (strand T2) and missing (strand T3) in the target strand comparing with the target strand T1 and blank control by the DNA dumbbell nanoswitch. Sequences of the strands are given above the bar graph for comparison. The concentrations of DNA carrier and target strands are 0.125 nM and 2.5 nM, respectively. Error bars in (c) and (d) represent the standard error of the mean obtained from three repeated measurements (Table S5). (e) Atomic force microscopy (AFM) image of DNA carriers with target T1 in “open” state (top) and “close” state (bottom).

Here, we establish a nanopore sensor based on DNA dumbbell nanoswitches for multiplex nucleic acid detection and bacterial identification. Two groups of DNA dumbbells are placed next to the two sensing overhangs of the nanoswitch on the carrier after self-assembly (Figure 1a), and they can be drawn together when the loop is formed upon target strand binding (Figure 1b). Translocating through glass nanopores (pore diameter ∼14 nm), the dumbbells on the carrier mark the sensing site by inducing double peaks in the ionic current signal in absence of the target. Although the resolution of the large glass nanopores is not as high as the recently reported small glass nanopores,^42^ larger nanopores are much easier to fabricate and handle as well as more suitable for long measurement times with complex biologic samples.^38,43^ Due to the extended sensing region of our 14 nm large glass nanopores, the two peaks create a much larger current drop when the DNA dumbbells are drawn close to each other by binding to the target strand. The key improvement to other nanopore sensing techniques is the precise analysis of its corresponding sensing site. The presence and change of the peaks due to the dumbbell marks avoid false positive or negative results. A thresholding algorithm can distinguish the “2”-peak from the “1”-peak, greatly simplifying data analysis. Based on this sensing strategy, we designed multiple DNA dumbbell nanoswitches on carriers to detect different DNA or RNA targets simultaneously even at single-base resolution. The fragments of 16S rRNA were also detected for bacterial identification. The programmable nanopore sensing platform has high flexibility and specificity in various nucleic acid sensing scenarios.

## Results and Discussion

### Single Target Detection by DNA Dumbbell Nanoswitch

We assessed the performance of our DNA dumbbell nanoswitch-nanopore sensor by detecting a short ssDNA (22 nt). The self-assembly of a DNA carrier with a dumbbell nanoswitch in the center is illustrated in Figure 1a, and design of the staples is presented in Figure S1a. Glass nanopores (∼14 nm) were utilized to monitor the DNA topology change. During the translocation, the long dsDNA induced a first level current drop *I*_0_ and the DNA nanostructures on the carrier triggered the secondary current drop Δ*I* (Figure S2). The appearance time of the secondary current drop closely matches the position of the nanostructure on the carrier. Based on the spatial resolution of our ∼14 nm glass nanopore, a gap of 178 bp was introduced between the two sensing overhangs to separate the two groups of DNA dumbbells. As expected, we obtained the double peak current drop signal in the “open” state (Figure 1b). After the addition of the target DNA strand T1 (Table S1), it hybridized with the two sensing overhangs, formed the loop, and drew the two groups of dumbbells together. This topology change resulted in the fusion of the two current drops into a single large downward peak into the “close” state. The length of the loop represents a compromise between the current drop, size of the loop, and distance of the dumbbells and ensures that the whole structure can pass through the nanopore (Figure S3). In this way, the small target binding was translated into the nanopore current signal change (Figure 1b and Figure S9). Even though the loop of the DNA nanoswitch is detectable by itself using the glass nanopore (Figure S4), the DNA dumbbells mark the sensing site on the carrier to avoid false positives, such as a secondary current drop caused by the DNA knot.^44^ More DNA also enhances the mean value of the relative current intensity (Δ*I*/*I*_0_) aiding peak identification. The signal switch in the current trace from a small double peak to a large single peak in the presence of a target can be directly recognized, thus greatly simplifying the data analysis.

We introduce the single peak fraction (SPF) to detect our nucleic acid targets. SPF is the ratio of the number of translocation events with a single peak to the total number of analyzed translocation events (SI 1.6). As shown in Figure 1c, the SPF keeps increasing with target concentration until reaching a plateau at 2.5 nM (ratio for concentration of target to carrier is 20). The limit of detection is ∼55 pM determined by 3 times the standard deviation of the blank. The sensitivity can be further improved by decreasing the concentration of the DNA carrier (Figure S8), but the extension of the measuring time must be considered at the same time.

We examined the specificity of our sensor by adding DNA targets with a single base substitution (strand T2, Table S1) or missing a single base (strand T3, Table S1). When mixed with the same carrier (Figure 1d) their SPFs (0.076 for T2 and 0.11 for T3) are much lower than for T1 (0.68) and close to the blank (0.045). Single base substitutions at different positions were also investigated, and the result is shown in Figure S5. The short length of the sensing overhang (11 nt) and tension in the loop are key reasons for the high specificity of the molecular switch. As shown by our results, the loop tends to open if the target is not completely complementary to the sensing overhangs. An AFM image of the carrier with DNA dumbbell nanoswitch in both states is shown in Figure 1e.

### Multiplexed Sensing via DNA Dumbbell Nanoswitches

**Figure 2:**
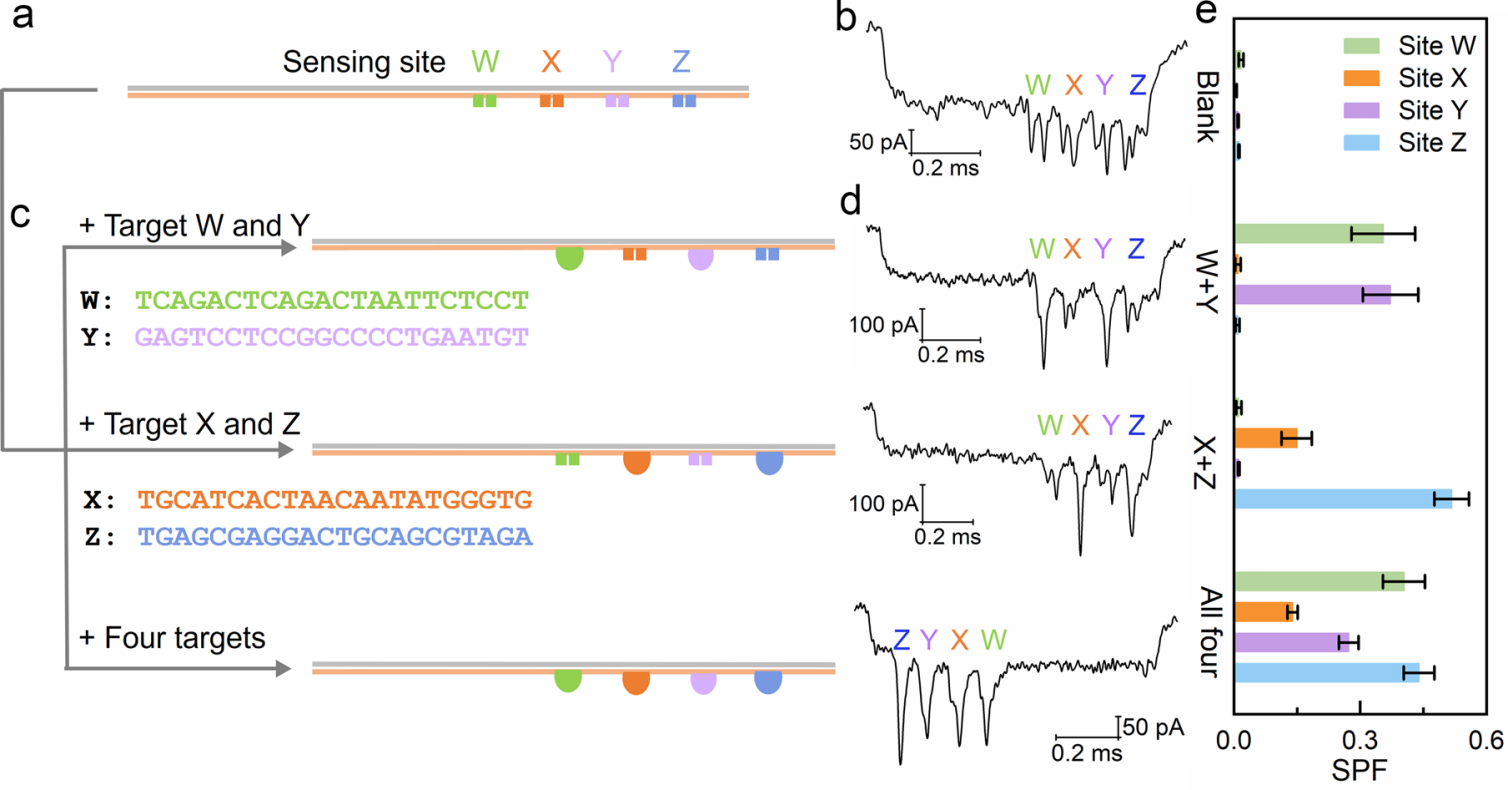
Multiplexed sensing of four different DNA target sequences by a carrier with four DNA dumbbell nanoswitches. (a) Sketch of a DNA carrier with four binding sites for target sequences W, X, Y, and Z. (b) A typical unfolded event showing four double peak patterns for the “blank” carrier without target. (c) From top to bottom: addition of strands W and Y, X and Z, and all four with corresponding ionic current signals in (d). Please note that the example signal for all four shows a carrier translocation in the opposite direction. The concentrations of DNA carrier and target strands are 0.125 nM and 2.5 nM, respectively. (e) Bar chart showing the SPF of the experiments from (a) and (c). Error bars represent the standard error of the mean obtained from three repeated measurements (Table S6).

As we have established that we can detect a single nanoswitch on a DNA carrier we now show that we can add four nanoswitches on a single carrier. With a single carrier, simultaneous detection of multiple targets by designing several DNA dumbbell nanoswitches at different positions was realized. As schematically shown in Figures 2 and S1b, four dumbbell nanoswitches were placed at one side of the carrier with equivalent space between them to detect the four DNA target sequences W, X, Y, and Z (Table S1). The basic rules followed in choosing the target sequences include maintaining a GC content between 30% and 70%, avoiding the region of continuous G or C repeat and a highly folded region such as the stem of the hairpin. Four successive double peaks marking the four dumbbell nanoswitches were observed as the example event shown in Figure 2b when the carrier passed through the aperture without any targets (blank). Samples containing different combinations of the four targets were tested using the carrier with four dumbbell nanoswitches. When targets W and Y or X and Z were present in the sample, single large peaks are observed at the expected sites (Figure 2c,d). When all four target strands were added, all 4 sites can be occupied as shown in the example in the lower panel in Figure 2c,d.

For the detection we compare the SPFs in Figure 2e for the blank and occupied carriers. The false positive rates at the four sensing sites were controlled at SPF < 0.017 ± 0.006 (site W, the highest one) as shown in the top bar graph of the blank sample in Figure 2e. The sensing sites were easily identified by their distinctive double-peak patterns. Any false signals of randomly occurring current drops caused by background noise or DNA knots on the carrier^44^ are avoided. Moreover, the reference structures that were used at the two ends of the sensing area in our previous work^36−38^ are not needed. The double-peaks also reduce the impact of the velocity fluctuation and variation during DNA translocation^40,41^ on signal identification.

In contrast to the blank data, the SPFs are found to increase to around 0.3 after strands W and Y are added. Importantly, The SPF for X and Z remained at less than 0.01, Table S6. Similarly, single large peaks were also observed at sensing site X and Z when the corresponding targets were present in the sample. Here the SPF for W and Y is again below 0.01. Finally, all four targets were added, and SPFs at all four sensing sites exceeded 0.15 (Figure 2e, bottom row). More example events are shown in Figure S10. Our results show that the nanopore-based dumbbell nanoswitches can be used for multiplex detection of DNA sequences. Furthermore, this sensing method should work for various single-stranded DNA and RNA from biological samples with the appropriate sequence and length.

It is interesting to note that the SPF of target X is similar for both the two and four target cases, showing that cross-binding is not significant. Notably, the SPF at site X is lower than the other sites, which could be associated with the molecular internal interaction (self-folding) between the overhang and scaffold binding domain of the probe strands (Figure S6). Computer-assisted structure prediction and optimization could be applied in the future to prevent this issue at the design stage.

Direct detection of viral RNA is also possible using the DNA dumbbell nanoswitch. Different concentrations of MS2 RNA at the nanomolar level can be detected by DNA carriers with its corresponding nanoswitch probes after fragmentation (Figure S7). SARS-CoV-2 synthetic RNA (880 nt) with a Cq value of 21 was detected (Figure S8), but enrichment of the SARS-CoV-2 sample and a decrease in the carrier concentration are needed currently for the PCR-free virus detection method. Even with a single solid-state nanopore we had to measure for more than 10 h at picomolar DNA concentration until we obtained the 10–20 events required for a positive result.^33^ The future development of parallel nanopore measuring systems will increase the throughput and will be helpful for applications for analysis of clinical samples at picomolar concentrations.

### Identification of Single Base Variation via Barcoded Carriers

**Figure 3:**
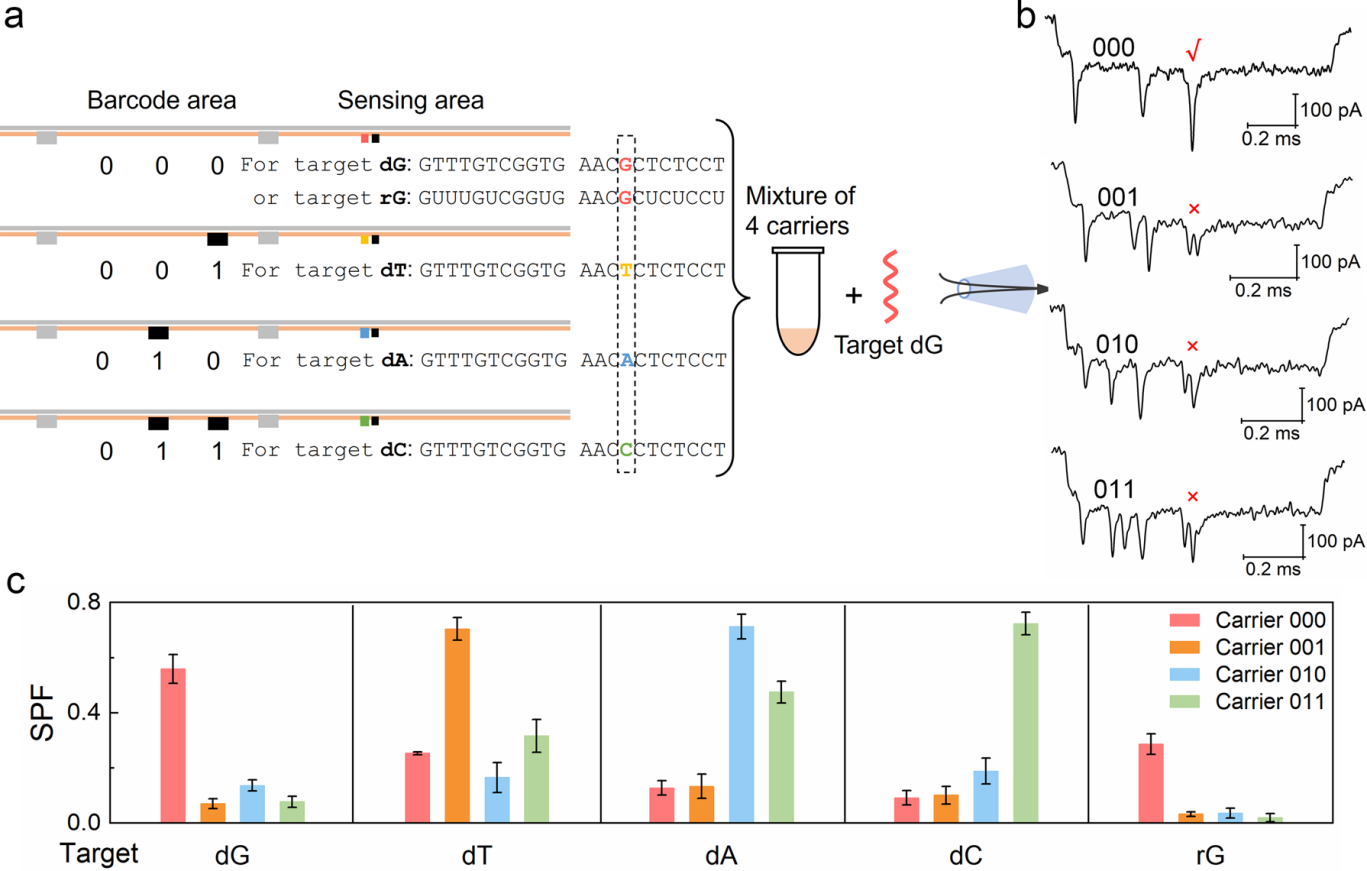
Identification of single base variation by DNA dumbbell nanoswitch-nanopore sensor. (a) Schematic of four barcoded carriers with a single DNA dumbbell nanoswitch as indicated in the sketch. We designed four carriers with barcodes 000, 001, 010, and 011 to test the effect of the four dG, dT, dA, and dC, respectively. The position of the base in the target strand is indicated by the dashed box. The RNA target rG can also be detected by carrier 000. (b) Detection of dG is shown as an example and the example events of the four carriers are given with only the carrier 000 showing a large single downward peak. (c) SPFs of each carrier obtained from different targets. The concentrations of DNA carrier and target strands are 0.125 nM and 2.5 nM, respectively. Error bars represent the standard error of the mean obtained from three repeated measurements (Table S7).

We expected that our DNA dumbbell nanoswitch should be sequence specific (Figure 1d) and capable of identifying single nucleotide substitution. Specifically, we studied whether our DNA nanoswitch assay can be used not only to detect point mutations^45−47^ but also to report what base the single nucleotide is changed at the mutation site. To this end, four carriers with different barcodes were designed on one side of the DNA carrier using the dumbbell-based encoding system (Figure 3a, S1c, Table S2),^35^ to enable simultaneous detection and classification of DNA carriers. In the sensing area, two short DNA overhangs were placed at the same sensing site along each of the four DNA carriers, with every pair of overhangs designed to efficiently bind a different DNA target with a specific base difference. The use of different carriers avoids crosstalk between different sensing sites on the same carrier; e.g., a target strand hybridizes with two overhangs from two different nanoswitches, because the four dumbbell nanoswitches share the same sequence for one of the sensing overhangs (illustrated in black in Figure 3). Choosing the same sensing site on different carriers avoids the potential impact of different scaffold binding sequences on SPF and ensures that the four dumbbell nanoswitches will work in the same conditions; hence, the comparison of SPFs between the four dumbbell nanoswitches is more meaningful.

We demonstrate the detection accuracy of our four DNA carriers by mixing one type of target DNA strands (i.e., with only one type of base mutation) with a mixture of four different carriers. For instance, Figures 3a, b and S11 show the detection of dG DNA target only by the appropriate carrier 000 (see Table S1), while the other carriers show negligible binding to the same target (Figure 3c, first group). Similarly, a selective detection of all other three targets was observed when separately added to the carrier mixture, as indicated by the high SPF values (Figure 3c, Table S7). The highest SPF was always obtained from the correct carrier designed for the corresponding target. The false signal at carrier 011 in the presence of target dA may be due to the stable structure formed by the target and sensing overhang of carrier 011 (Figure S12), which could be improved by adjusting the design and choosing the suitable target domain with computer-aided optimization in the future. These results show that the DNA dumbbell nanoswitch-based multiplex sensing system can identify the single base variation. Furthermore, RNA target rG with an equivalent sequence as dG was also detected by this nanopore sensor and induced the highest SPF on carrier 000 as expected (Figure 3c, last group). The positive detection indicates that the nanopore sensor can also be used for RNA detection.

### Bacterial Identification via Barcoded Carriers with Multiple Dumbbell Nanoswitches

**Figure 4:**
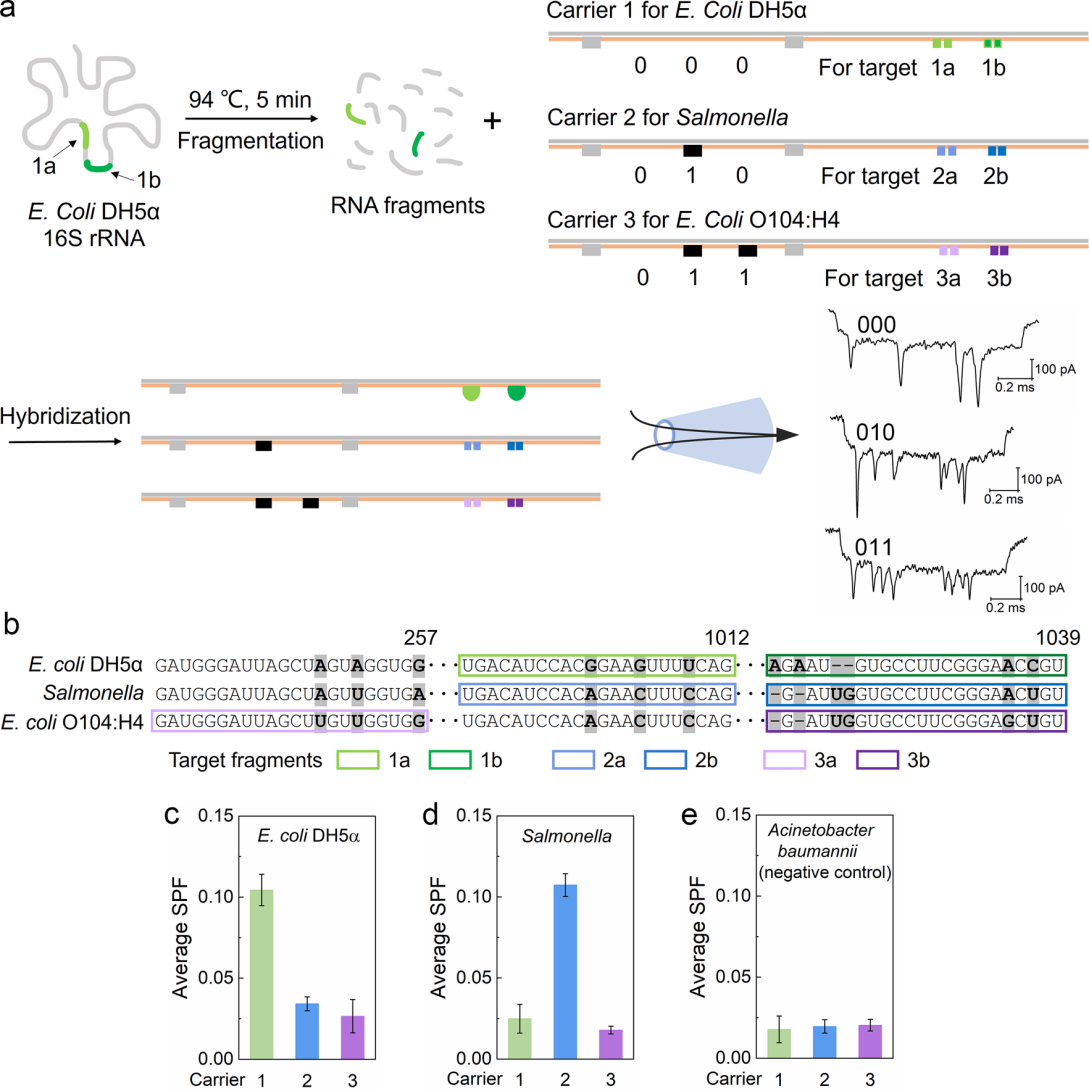
Bacterial identification by detection of 16S rRNA fragments using barcoded carriers with multiple dumbbell nanoswitches. (a) Schematic of the detection of *E. coli* DH5α 16S rRNA fragments. Two DNA dumbbell nanoswitches are located at two sensing sites on each carrier to detect two target RNA fragments from the same bacterium. Carrier 1, 2, and 3 are designed for *E. coli* DH5α, *Salmonella*, and *E. coli* O104:H4, respectively. (b) Target rRNA fragments highlighted by colored frames from 16S rRNA of the three bacterial species for identification. (c–e) Average SPFs of the two sensing sites on each carrier obtained from different 16S rRNA fragments. Results of *E. coli* DH5α, *Salmonella*, and *Acinetobacter baumannii* (negative control) are given in (c), (d), and (e), respectively. Error bars for the standard error of the mean in (c), (d), and (e) are based on three repeated measurements (Table S8).

The high single-base specificity of the DNA dumbbell nanoswitch-nanopore sensor for RNA targets generates a method for identifying bacteria through detecting their 16S rRNA.^48−50^ As a proof of principle, three digital encoded carriers with multiple dumbbell nanoswitches (Figures 4a, S1d) were designed to detect the target sequences from 16S rRNAs of bacterial species *E. coli* DH5α, O104:H4, and *Salmonella* (Figure 4b). The different barcodes loaded on the three carriers are used to identify current signals and make the designs asymmetric to determine translocation direction; hence, multiple carriers for different 16S rRNAs were measured simultaneously. These three bacterial species share a high degree of similarity in their 16S rRNA sequences; hence, it is an ideal model to examine the ability of the nanopore sensor for differentiating RNA sequences from complex biological samples. To improve the accuracy of bacterial identification, two target fragments of 16S rRNA were selected for each bacterial species and two dumbbell nanoswitches were designed for them on each carrier.

Given the length of 16S rRNA and the folded structure of long RNA, the total RNA extracted from bacteria was first fragmented into pieces by incubation in RNA fragmentation buffer at 94 °C for 5 min.^21^ Then the three DNA carriers were mixed with the RNA fragments to recognize the target sequences. A case study of *E. coli* DH5α is shown in Figures 4a and S13. Large single peaks were observed at the two sensing sites on carrier 1 as the example event shows in Figure 4a. We used the average SPF (SI 1.6) of the two dumbbell nanoswitches on each carrier to identify the specific bacterial species. The highest average SPF was obtained from carrier 1 (Figure 4c) by testing the total RNA of *E. coli* DH5α, because the sensing overhangs on carrier 1 were 100% complementary to the two target 16S rRNA fragments of *E. coli* DH5α. The generated SPF was lower than the ones collected from DNA or pure RNA targets in Figures 2 and 3. This result may be caused by the random fragmentation and self-folding of rRNA fragments of different lengths. A similar result was obtained on carrier 2 when testing the total RNA of *Salmonella* in the same way (Figure 4d). In contrast, the average SPF of dumbbell nanoswitches on carrier 3 was maintained at a low level (<0.05), even though the target sequences of *E. coli* O104:H4 are highly similar to the sequences from *E. coli* DH5α and *Salmonella*. As a negative control, *Acinetobacter baumannii* total RNA was also fragmented and mixed with the three carriers; no significant SPF was obtained from the six dumbbell nanoswitches (Figure 4e, Table S8). These data show that our dumbbell nanoswitch-based multiplex sensing assay identified the bacterial species by detecting their 16S rRNA fragments, even though their 16S rRNA sequences are highly similar (Figure 4b). Therefore, this approach offers a facile approach for rapid bacterial identification without PCR and sequencing avoiding fluorescent labels. The identification accuracy will be further improved by adding more target fragments and dumbbell nanoswitches to meet the requirement for more complex conditions.

## Conclusion

In conclusion, we have created a DNA dumbbell nanoswitch on the carrier and used glass nanopores to detect the topology change caused by target strand binding. In the “open” state of the switch, the two groups of DNA dumbbells induce a downward double peak in the current trace to precisely mark the position of the sensing site on the carrier, so the signal can be easily recognized by the unique shape instead of appearance time during the carrier translocation. Upon target strand binding, the sensor is switched into the “close” state, in which the loop is formed, and two groups of dumbbells are drawn together. The nanopore current signal is also switched from small double peak into a large single peak, which can even be directly recognized by eye. This feature decreases the false positive rate and simplifies data analysis, especially in multiplexed sensing. By placing four DNA dumbbell nanoswitches on one carrier, a multiplex nucleic acid sensing assay is established and four different target sequences were simultaneously detected. High specificity for the target sequence was another important feature of the nanopore sensor. To identify the point mutation in the target strand, digitally encoded carriers were applied to load different dumbbell nanoswitches with similar sensing overhangs at the same position of each carrier, so the crosstalk between the switches can be avoided and these switches are parallelly compared in the same situation. Nucleic acid targets with only one base difference can be successfully distinguished by this assay. Finally, three encoded carriers with two dumbbell nanoswitches on each were designed to identify bacteria species with high similarity by detecting their 16S rRNA fragments. The DNA dumbbell nanoswitch and barcoded carrier were rationally integrated together to realize the simultaneous detection of multiple nucleic acids with high specificity. *E. coli* DH5α and *Salmonella* were both correctly identified. In the future, the multiplexed sensing system may be expanded by adding more dumbbell nanoswitches on each carrier and updating the encoding system^33^ to measure more carriers simultaneously. Thus, more target fragments from a 16S rRNA could be selected to improve the accuracy of analysis and more pathogens and biomarkers can be analyzed.

The DNA dumbbell nanoswitch-nanopore sensor offers significant advantages over the existing methods. Compared to our previous nanopore-DNA carrier-based methods,^35,38,51^ the new multiplex assay does not need any modification and generates a positive feedback signal in the presence of target, and the unique double peak marked sensing site makes the data analysis much easier and more accurate. Compared to the previous nanopore sensing method used for DNA nanoswitches,^31^ the current method shows much higher potential for rapid and multiplexed sensing. Compared to the DNA nanoswitch based on gel readout,^9,18−21^ the DNA dumbbell nanoswitch is a single-molecule technique. In addition, nanopore analysis can provide more information with higher resolution^52,53^ and potentially faster per sample than gel electrophoresis. The nanopore translocation event can be collected at high frequency such as one event per second per nanopore for the development of a quick test. Multiple nanoswitches on the same carrier can be distinguished by their positions with the nanopore readout enabling multiplexing, which would be difficult to achieve with gel electrophoresis. Our digital encoding strategy enables simultaneous analysis of barcoded carriers with multiple dumbbell nanoswitches, greatly increasing the number of analytes. Utilizing the ability to use multiple target sequences based on the parallel analysis, bacterial strains with high similarity can be identified on different carriers in the same situation with less crosstalk. In summary, our DNA dumbbell nanoswitch-nanopore sensor has promising applications in the fields of bioanalysis, biomedicine, and clinical diagnostics.

## Supporting Information Available

Materials, experimental methods, sequences of nucleic acids, supplementary nanopore data, and figures can be found in the Supporting Information. (PDF)
