# Supplementary material for: Multiplexed Nanopore-Based Nucleic Acid Sensing and Bacterial Identification Using DNA Dumbbell Nanoswitches
Source: J Am Chem Soc. 2023 May 23;145(22):12115–23. doi: 10.1021/jacs.3c01649 (PMC10251517; doi:10.1021/jacs.3c01649)
Supplement: Supplementary file 1 — ja3c01649_si_001.pdf [file ja3c01649_si_001.pdf]

Supporting Information for

Multiplexed nanopore-based nucleic acid sensing and bacterial identification using DNA dumbbell nanoswitches

Jinbo Zhu,[a,c] Ran Tivony,[a] Filip Bošković,[a] Joana Pereira-Dias,[b] Sarah E. Sandler,[a] Stephen Baker,[b] Ulrich F. Keyser[a,*]

[a]Cavendish Laboratory, University of Cambridge, JJ Thompson Avenue, Cambridge, CB3 0HE, UK. E-mail: ufk20@cam.ac.uk

[b]Cambridge Institute of Therapeutic Immunology & Infectious Disease (CITIID), Jeffery Cheah Biomedical Centre, Cambridge Biomedical Campus, University of Cambridge, Cambridge, CB2 0AW, UK.

[c]School of Biomedical Engineering, Faculty of Medicine, Dalian University of Technology, No. 2, Linggong Road, Dalian, 116024, China

S1. Materials and experimental methods

S1.1 Materials

Oligonucleotides were purchased from Integrated DNA Technologies, Inc. (IDT). The sequences of DNA and RNA strands are listed in Table S1-3. M13mp18 ssDNA, RNA fragmentation buffer, BamHI-HF, and EcoRI-HF were purchased from New England Biolabs. MS2 RNA was purchased from Sigma-Aldrich. *E. coli* DH5α total RNA was purchased from Thermo Fisher Scientific. Synthetic SARS-CoV-2 RNA (880 nt, EURM-019) was purchased from European Commission, Joint Research Centre. *Salmonella* total RNA was extracted from bacterium cultured in laboratory. Other chemicals were of reagent grade and were used without further purification.

S1.2 Preparation of DNA carriers

190 DNA oligonucleotides (staples) designed in our previous work[1] were used here for M13 linearization. The staples were accurately mixed before preparation of the DNA carriers. Design of the carriers used in this work are shown in Figure S1. The staples at sensing sites are replaced with dumbbell and probe strands as listed in Table S1 and S3. For DNA carriers with barcodes, the staples at coding sites are replaced with dumbbell strands as listed in Table S2.

The carrier synthesis follows our previous work.[1] The 7228 nt DNA scaffold was linearized from M13mp18 ssDNA using the published protocol.[1] After mixing the staples of relevant carriers following the design in Figure S1 and Table S4, the cut M13 scaffold was added into the solution (20 nM M13 scaffold, 60 nM staples and 120 nM dumbbell strands or probes) and heated to 70°C followed by a linear cooling ramp to 25°C over 50 minutes. Finally, the resulting solution was diluted with a washing buffer (10 mM Tris-HCl, 0.5 $MgCl_2$, pH 8.0) to 500 μL and centrifuged with an Amicon Ultra 100kDa filter to remove the excess DNA strands at 6000 g for 10 mins (repeated 3 times). About 35 μL solution of DNA carrier was obtained and quantified with NanoDrop 2000 spectrophotometer.

S1.3 Fragmentation of 16S ribosome RNA (rRNA) and SARS-CoV-2 RNA

0.5 μL of *E. coli* DH5 total RNA (1.0 μg/μL), 0.5 μL of *Salmonella* total RNA (0.57 μg/μL), 0.5 μL of Acinetobacter baumannii total RNA (1.1 μg/μL), or 0.5 μL of MS2 RNA at different concentrations was mixed with 1 μL of 10× RNA fragmentation buffer, and then nuclease-free water was added to 10 μL. 30 μL of Synthetic SARS-CoV-2 RNA (880 nt, Cq value 21) was dried in vacuum concentrator (SpeedVac DNA 130, Thermo Fisher Scientific) and rehydrated with 9 μL nuclease free water before mixed with 1 μL of 10× RNA fragmentation buffer. The mixture was incubated in a preheated thermal cycler for 5 minutes at 94℃ then cooled down to 4℃. Finally, 1 μL of 10× RNA fragmentation stop solution and 0.5 μL of RNAsecure reagent were added into the mixture.

S1.4 Nanopore measurement

The fabrication and measurement of the glass nanopore follows the former study.[1] Glass nanopores with diameters 14±3 nm were generated on a laser-assisted pipette puller (P-2000, Sutter Instrument) by pulling quartz capillaries (outer diameter 0.5 mm and inner diameter 0.2 mm, Sutter Instrument). The resulting nanopores were fixed on a PDMS chip. I-V curve was measured in 4 M LiCl to check the size and current noise of nanopore before the addition of sample.

Target strand was incubated with 1 nM DNA carrier (concentration for each carrier) in TM buffer (10 mM Tris-HCl, 10 mM $MgCl_2$, pH 8.0) at room temperature for 10 minutes. For bacterial or MS2 RNA detection, 10 μL of the RNA fragments mixture was mixed with 5 μL of 1 nM DNA carrier (concentration for each carrier) and incubated at room temperature for 10 minutes. Then carrier solution was diluted with Tris-LiCl buffer solution (10 mM Tris-HCl, 4 M LiCl, pH 9.0) to 0.125 nM and then added to the tip side of the glass nanopore. Two electrodes were placed at the two sides of the nanopore. The electrodes were connected to an Axon Axopatch 200B amplifier (Molecular Devices), which applied a voltage of 600 mV to drive the DNA through nanopores and recorded the current signal. For 0.125 nM carrier, the measurement lasted for 2-3 hours, but more time (~12h) was needed for carrier at low 10s of picomolar level in Figure S8. The current signal was filtered with an external Bessel filter (Frequency Devices) at 50 kHz and digitized at a 250 kHz sampling rate with a data card (PCI-6251, National Instruments).

S1.5 Atomic force microscopy (AFM) imaging

DNA carrier investigated in Figure 1 with target T1 was diluted to 0.1 nM in TM buffer for AFM measurement. 10 μL of 0.1 nM carrier sample was dropped onto a freshly cleaved mica surface for 1 minute, rinsed three times with 100 μL of water, and dried gently by nitrogen flow. The mica plate was affixed to the AFM sample stage using double-sided adhesive tape before the scan. Gwyddion was used for image visualization and analysis.

S1.6 Nanopore data analysis

Home-made LabVIEW algorithms were used for data collection and analysis. The raw data was analyzed by following the steps described in the previous works[1,2]: (1) filter and search for the translocation events from the raw current trace; (2) remove the folded and uncertain events; (3) barcode and sensing peak determination. Single peak fraction (SPF) was calculated as below to indicate the presence of target sequence in the sample.

$$SPF = \frac{Number\ of\ events\ with\ single\ peak\ at\ sensing\ site}{Total\ number\ of\ events\ with\ double\ and\ single\ peaks}$$

Average SPF was used for bacterial identification. It is the average of the SPFs of the two sensing sites on the same carrier in Figure 4.

S2. Figures

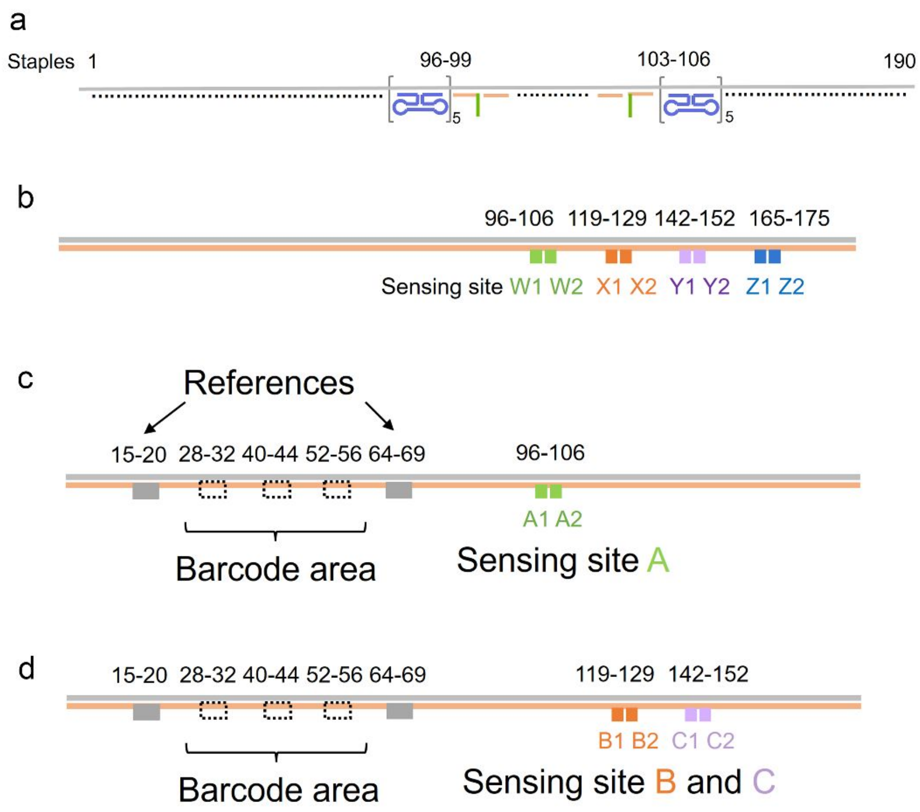

Figure S1. Design of carriers used in this work. (a) is for Figure 1, (b) for Figure 2, (c) for Figure 3, and (d) for Figure 4. 190 staples are used to linearize the M13 scaffold. Some staples labeled above the carrier are replaced with DNA dumbbells or probes as listed in Table S4.

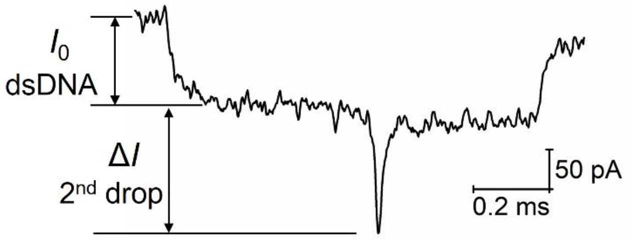

Figure S2. Definition of first level current drop I_0 and second level current drop ΔI shown by a translocation event.

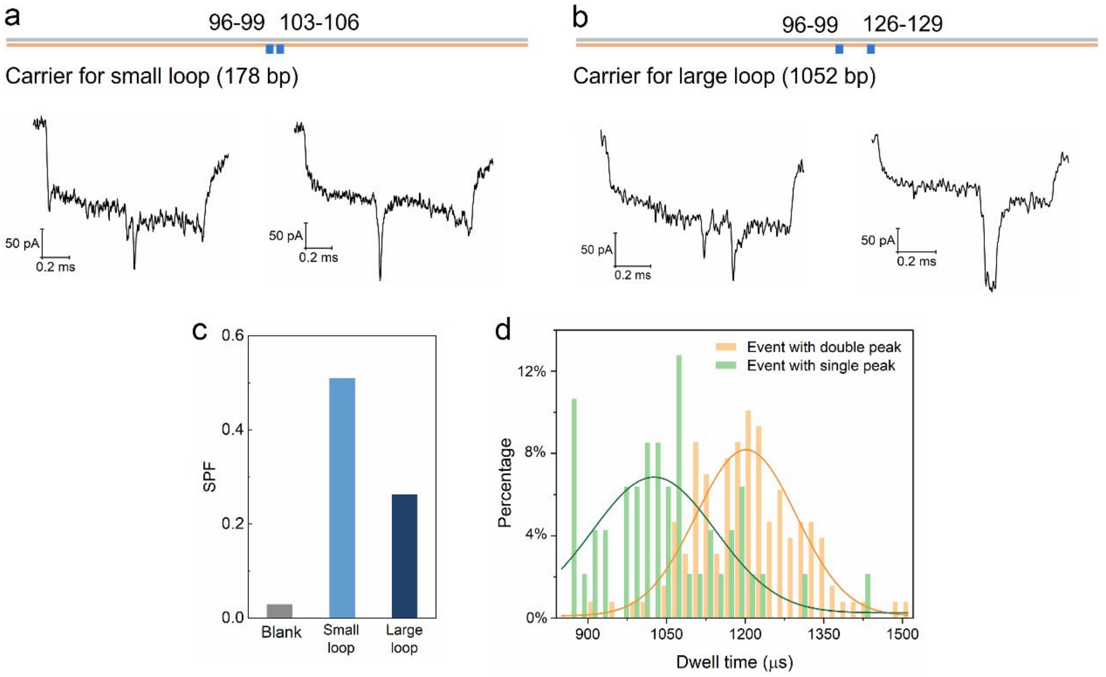

Figure S3. Comparison of DNA dumbbell nanoswitches with (a) small loop (178 bp) and (b) large loop (1052 bp). The concentrations of DNA carrier and target strands are 0.125 nM and 2.5 nM, respectively. Example events with single or double peak are shown in (a) and (b). (c) SPFs of blank sample and two carriers with the same concentration of target M. (d) Comparison of the dwell time of translocation events with (green) and without (orange) large loop. The analysis was based on 47 events with single peak and 129 events with double peak. They were recorded in the same measurement.

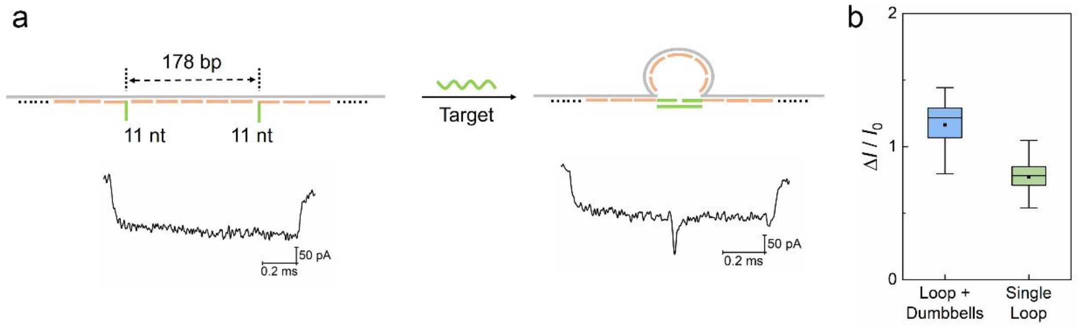

Figure S4. Nanopore detection of DNA target using DNA nanoswitch without DNA dumbbells. (a) Scheme and example events of the DNA nanoswitch-based DNA detection readout by glass nanopore. (b) Comparison of relative single-peak intensities ($\Delta I/I_0$) between carriers with dumbbells and without dumbbells (Single Loop) in presence of target T1. The concentrations of DNA carrier and target strand are 0.125 nM and 2.5 nM, respectively. The analysis is based on 104 events for carriers without dumbbells and 137 events for carriers with dumbbells. The mean value is represented by a line across the box and median is represented by a dot in the box.

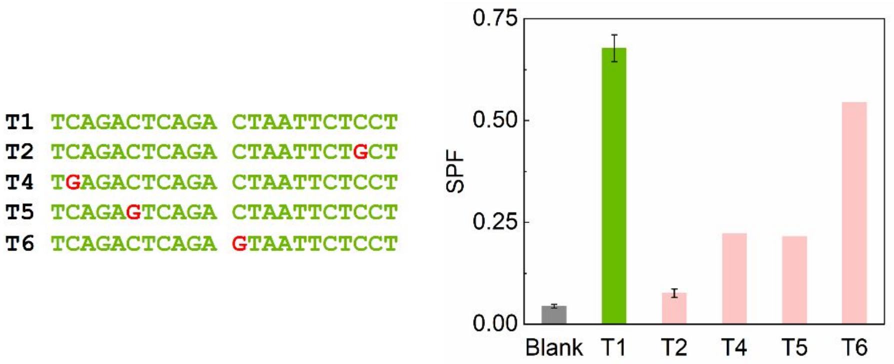

Figure S5. Effect of single base substitution at different positions of the target sequence on the SPF of the carrier in Figure 1. The result indicates that mismatches at different positions show different effects on the SPF. Mismatches that are close to the two ends of the target decrease the SPF much more than the mismatch in the middle of the target.

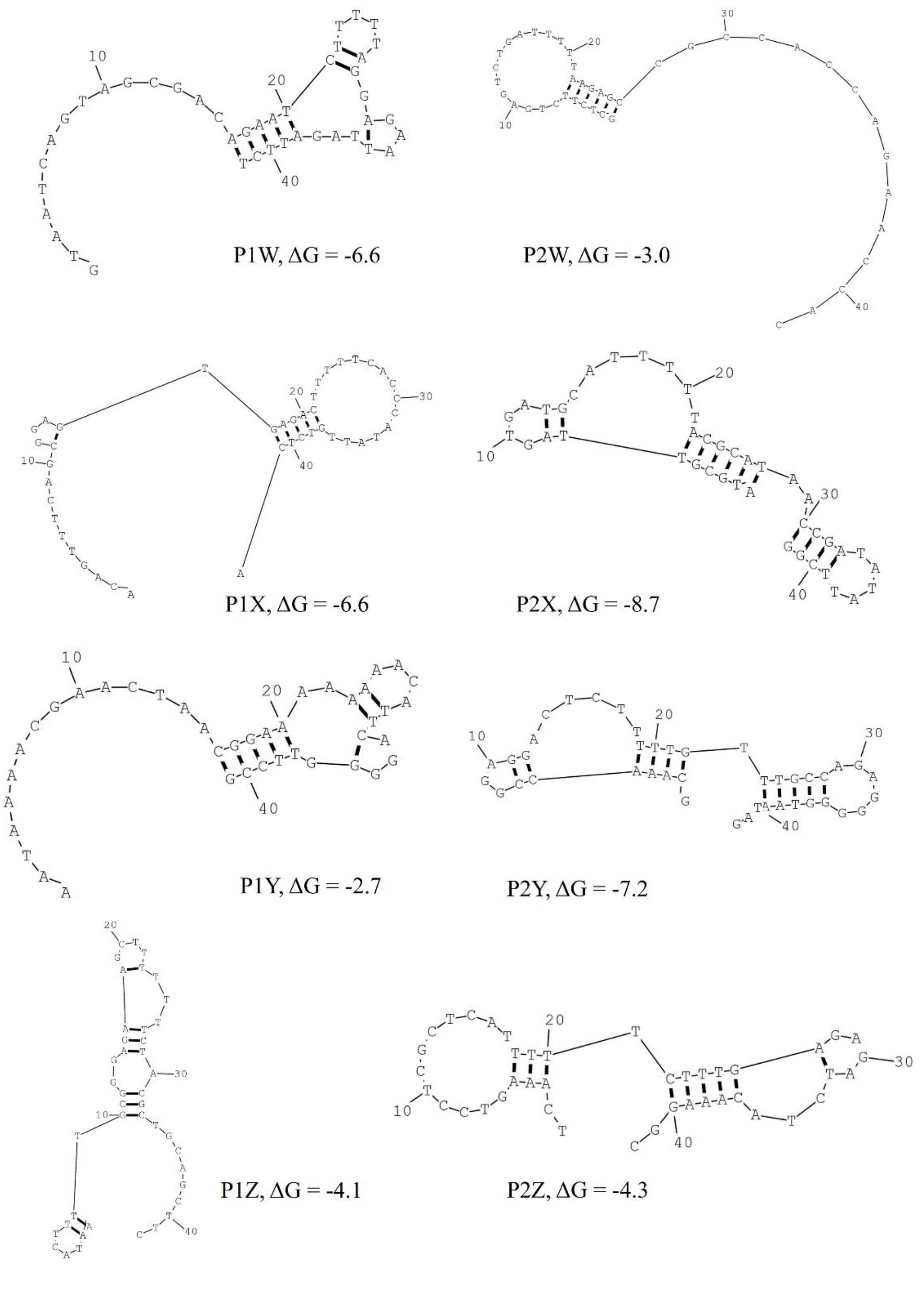

Figure S6. Self-folding energy of probe strands (Table S1) predicted by RNAstructure (version 4.6). P2X at sensing site X2 has the most stable self-folding structure, which will affect its binding to M13 scaffold and target.

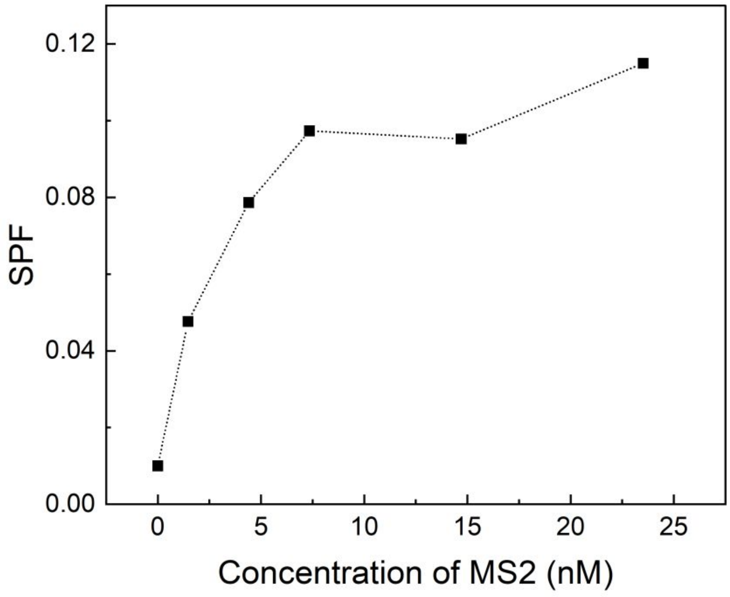

Figure S7. Detection of MS2 viral RNA after fragmentation at different concentrations using DNA dumbbell nanoswitch. 0.125 nM carrier is used in all measurements. More detailed nanopore data can be found in Table S9.

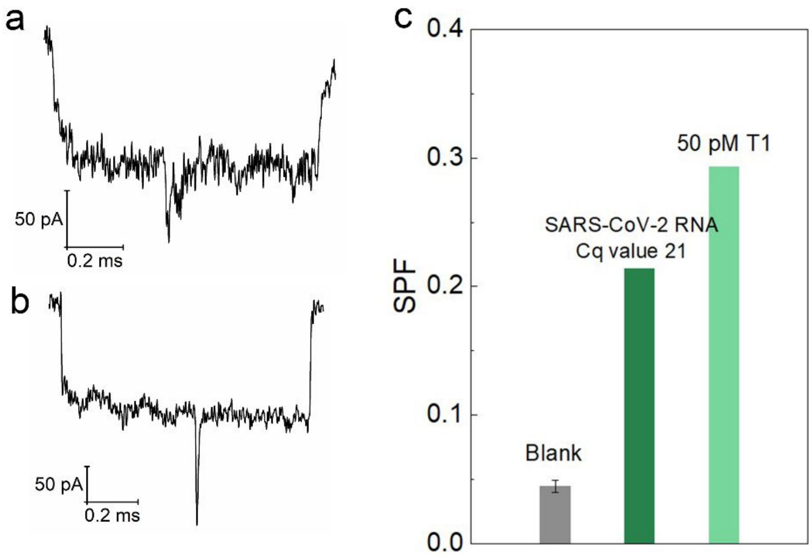

Figure S8. Detection of target T1 at 50 pM and synthetic SARS-CoV-2 RNA (880 nt) with Cq value at 21 using single DNA dumbbell nanoswitch on carrier shown in Figure 1. Example events without target and with target are given in (a) and (b), respectively. (c) SPFs of blank, SARS-CoV-2 RNA, and 50 pM T1. 28 translocation events (6 events with single peak) and 58 translocation events (17 events with single peak) were analyzed to calculate the SPFs of SARS-CoV-2 RNA and T1, respectively. 5.0 pM carrier was used for 50 pM T1 and 2.2 pM carrier was used for SARS-CoV-2 RNA. SARS-CoV-2 RNA was concentrated 3.3 times and fragmented in RNA fragmentation buffer before mixed with carrier.

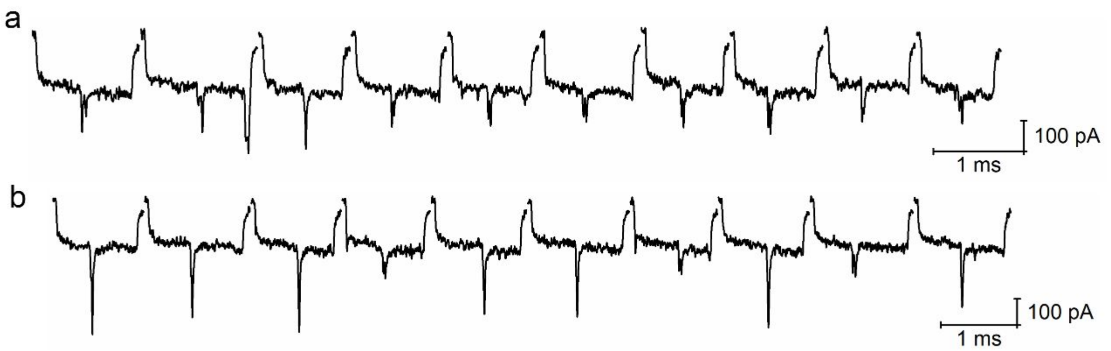

Figure S9. First ten translocation events of DNA carrier in Figure 1b (a) without target and (b) with target T1.

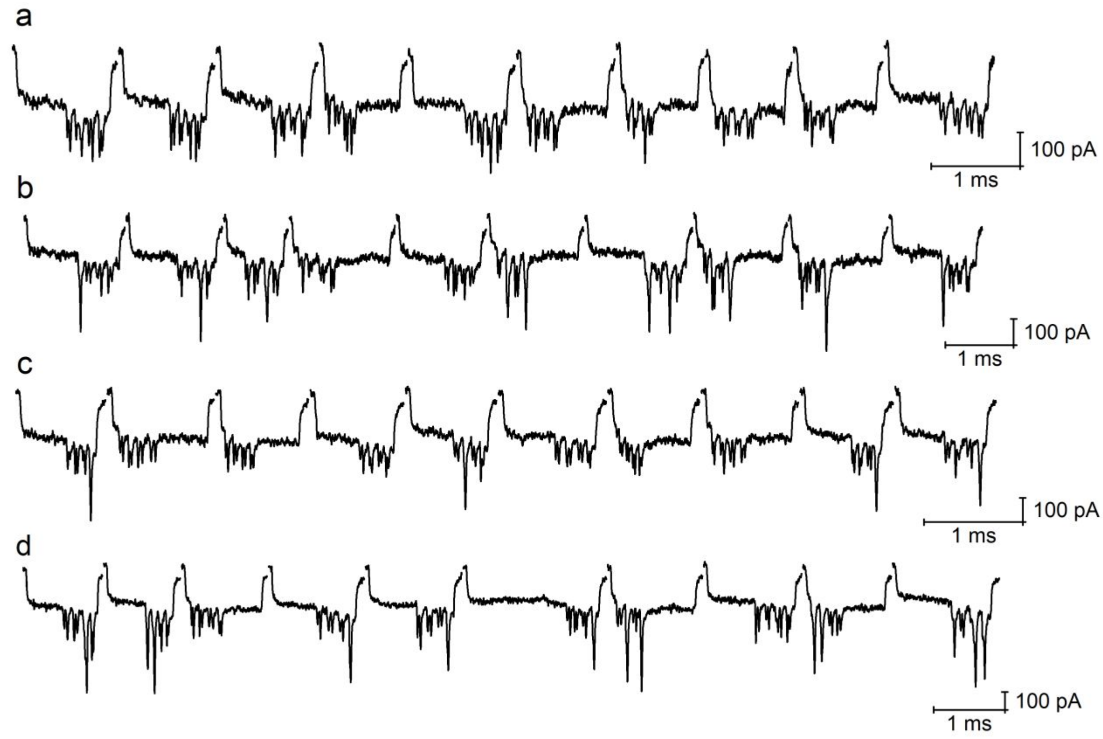

Figure S10. First ten translocation events of DNA carrier in Figure 2 (a) without any target, (b) with target W and Y, (c) with target X and Z, and (d) with all four targets.

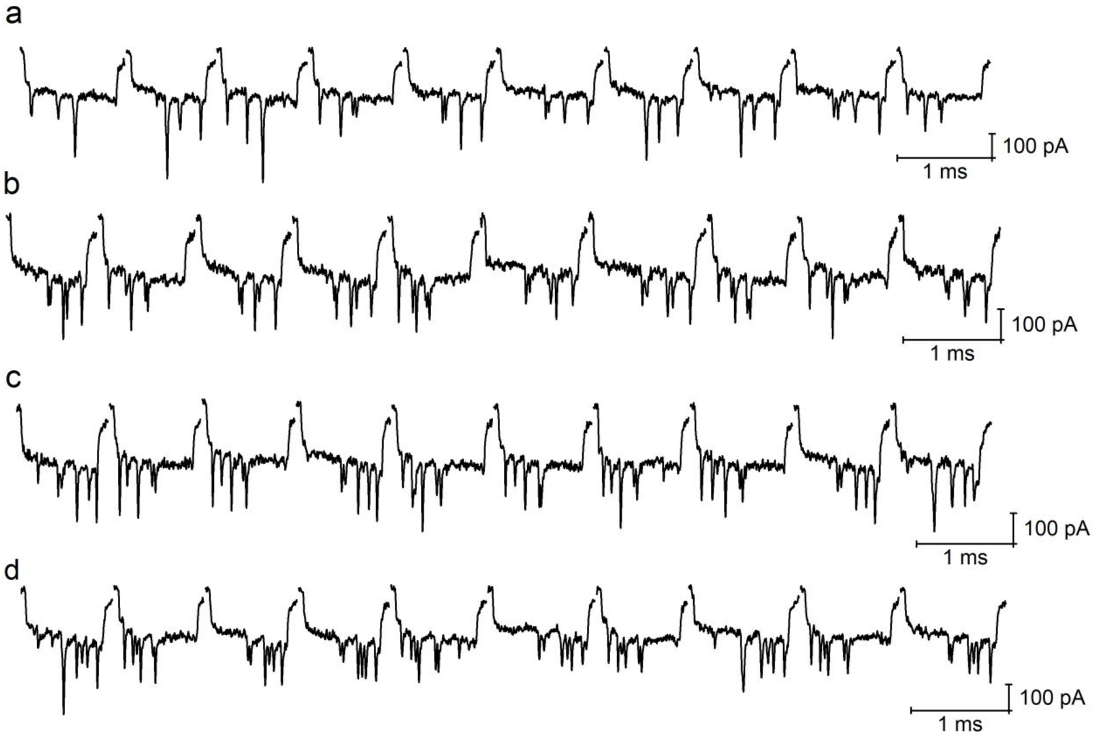

Figure S11. First ten translocation events of DNA carriers in Figure 3 with target dG. The events are classified into four groups, (a) 000, (b) 001, (c) 010, and (d) 011, by the barcodes.

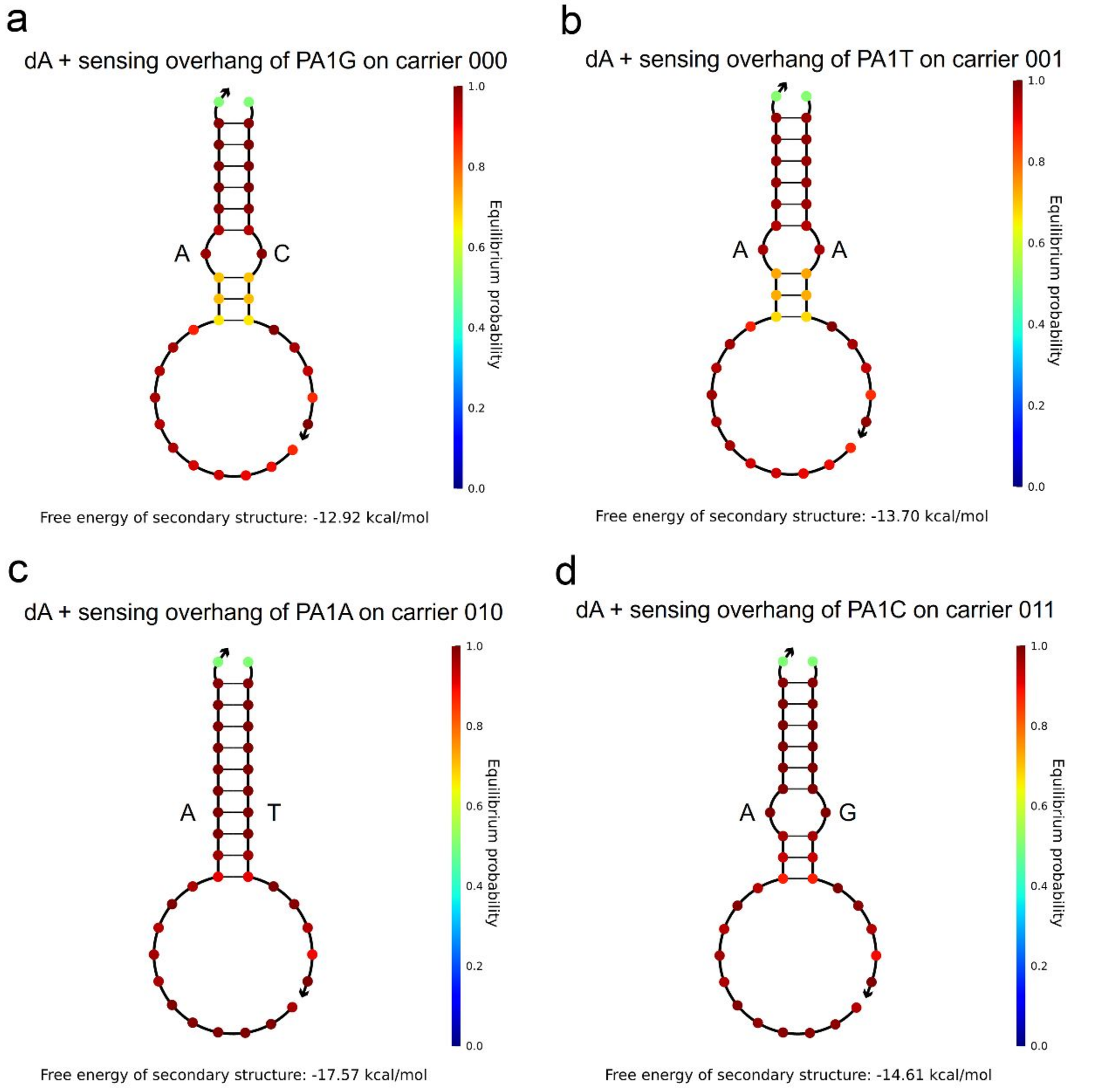

Figure S12. Comparison of the stability of structures formed by target strand dA and overhangs at sensing site A1 on different carriers. Secondary structures at 25℃ and their free energy are generated at NUPACK's website (https://old.nupack.org).

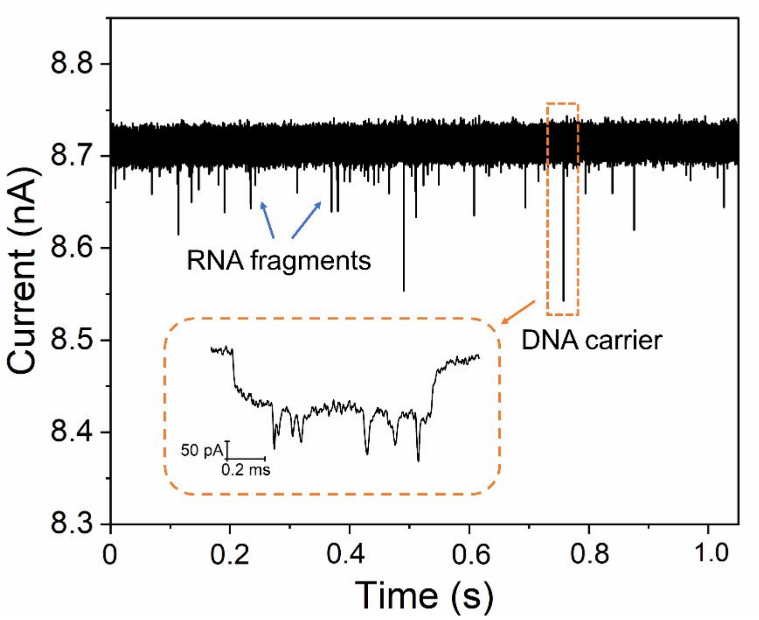

Figure S13. Raw current data collected from DNA carriers in Figure 4 with *E. coli* 16S rRNA fragments. The translocation event of carrier 2 was cropped and enlarged in the orange box. The translocation of RNA fragments can also be observed in the current trace.

S3. Tables

Table S1. Sequences of target nucleic acids and probes.[a]

Strand	Function	Sequence
T1/W	Target sequence	TCAGACTCAGA CTAATTCTCCT
T2	Single base substitution	TCAGACTCAGA CTAATTCTGCT
T3	Single base missing	TCAGA_TCAGA CTAATTCTCCT
T4	Single base substitution	TGAGACTCAGA CTAATTCTCCT
T5	Single base substitution	TCAGAGTCAGA CTAATTCTCCT
T6	Single base substitution	TCAGACTCAGA GTAATTCTCCT
P1W	Probe at W1 for T1/W	GTAATCAGTAGCGAC AGAATC TTTTT AGGAGAATTA GATTCT
P2W	Probe at W2 for T1/W	GCTCTT CTGAGTCTGA TTTTT AAGAGC CGCCACCAGAACCAC
X	Target sequence	TGCATCACTAA CAATATGGGTG
P1X	Probe at X1 for X	ACAGTTTCAGCGGAG TGAGAC TTTTT CACCCATATT GTCTCA
P2X	Probe at X2 for X	ATGCGT TAGTGATGCA TTTTT ACGCAT AACCGATATATTCGG
Y	Target sequence	GAGTCCTCCGG CCCCTGAATGT
P1Y	Probe at Y1 for Y	AATAAAACGAACTAA CGGAA AAAAA ACATTCAGGGG TTCCG
P2Y	Probe at Y2 for Y	GCAAAC CGGAGGACTC TTTTT GTTTGC CAGAGGGGGTAATAG
Z	Target sequence	TGAGCGAGGAC TGCAGCGTAGA
P1Z	Probe at Z1 for Z	AATACTTTTGCGGGA GAAGCT TTTTT TCTACGCTGC AGCTTC
P2Z	Probe at Z2 for Z	TCAAAG TCCTCGCTCA TTTTT CTTTGA GAGATCTACAAAGGC
dG	Target	GTTTGTCGGTG AACGCTCTCCT
rG	Target	GUUUGUCGGUG AACGCUCUCCU
dT	Target	GTTTGTCGGTG AACTCTCTCCT
dA	Target	GTTTGTCGGTG AACACTCTCCT
dC	Target	GTTTGTCGGTG AACCCTCTCCT
M	Target	ACCTGTAGCGT TCGTCAGAGCT
PA1G	Probe at A1 for dG/rG	GTAATCAGTAGCGACAGAATA AAAAA AGGAGAGCGT TATTCT
PA1T	Probe at A1 for dT	GTAATCAGTAGCGACAGAATA AAAAA AGGAGAGAGT TATTCT
PA1A	Probe at A1 for dA	GTAATCAGTAGCGACAGAATA AAAAA AGGAGAGTGT TATTCT
PA1C	Probe at A1 for dC	GTAATCAGTAGCGACAGAATA AAAAA AGGAGAGGGT TATTCT
PA2F	Probe at A2 for four	GCTCT CACCGACAAAC TTTTT AGAGC CGCCACCAGAACCAC
PB1a1	Probe at B1 for d1a	ACAGTTTCAGCGGAG TGAGA TTTTT CTGAAAACTTC TCTCA
PB2a1	Probe at B2 for d1a	ATGCG CGTGGATGTCA TTTTT CGCAT AACCGATATATTCGG
PC1b1	Probe at C1 for d1b	AATAAAACGAACTAA CGGAA AAAAA ACGGTTCCCGA TTCCG
PC2b1	Probe at C2 for d1b	GCAAA AGGCACATTCT AAAAA TTTGC CAGAGGGGGTAATAG
PB1a2	Probe at B1 for d2a	ACAGTTTCAGCGGAG TGAGA TTTTT CTGGAAAGTTC TCTCA
PB2a2	Probe at B2 for d2a	ATGCG TGTGGATGTCA TTTTT CGCAT AACCGATATATTCGG
PC1b2	Probe at C1 for d2b	AATAAAACGAACTAA CGGAA AAAAA ACAGTTCCCGA TTCCG
PC2b2	Probe at C2 for d2b	GCAAA AGGCACCAATC AAAAA TTTGC CAGAGGGGGTAATAG
PB1a3	Probe at B1 for d3a	ACAGTTTCAGCGGAG TGAGA TTTTT CCACCAACAAG TCTCA
PB2a3	Probe at B2 for d3a	ATGCG CTAATCCCATC TTTTT CGCAT AACCGATATATTCGG
PC1b3	Probe at C1 for d3b	AATAAAACGAACTAA CGGAA AAAAA ACAGCTCCCGA TTCCG
PC2b3	Probe at C2 for d3b	GCAAA AGGCACCAATC AAAAA TTTGC CAGAGGGGGTAATAG

PA1M	Probe at A1 for M	GTAATCAGTAGCGACAGAATT AAAAA AGCTCTGACG AATTCT
PA2M	Probe at A2 for M	GCTCT ACGCTACAGGT TTTTT AGAGC CGCCACCAGAACCAC
PB2M	Probe at B2 for M	ATGCG ACGCTACAGGT TTTTT CGCAT AACCGATATATTCGG

[a] Probe strands are folded into hairpin structures and the hairpins will be opened after binding with M13 scaffold. This design can avoid the hybridization between target and free probe strand in solution.

Table S2. Sequences of dumbbells used at barcode area.[a]

Strand	Sequence
Staple 15-20 Substitutes	
	TTTTTGGGGT TCCTCTTTTGAGGAACAAGTTTTCTTGT CGAGGTGCCG
	TAAAGCACTA TCCTCTTTTGAGGAACAAGTTTTCTTGT AATCGGAACC
	CTAAAGGGAG TCCTCTTTTGAGGAACAAGTTTTCTTGT CCCCCGATTT
	AGAGCTTGAC TCCTCTTTTGAGGAACAAGTTTTCTTGT GGGGAAAGCC
	GGCGAACGTG TCCTCTTTTGAGGAACAAGTTTTCTTGT GCGAGAAAGG
	AAGGGAAGAA TCCTCTTTTGAGGAACAAGTTTTCTTGT AGCGAAAGGA
	GCGGGCGCTA TCCTCTTTTGAGGAACAAGTTTTCTTGT GGGCGCTGGC
	AAGTGTAGCG TCCTCTTTTGAGGAACAAGTTTTCTTGT GTCACGCTGC
	GCGTAACCAC TCCTCTTTTGAGGAACAAGTTTTCTTGT CACACCCGCC
	GCGCTTAATG TCCTCTTTTGAGGAACAAGTTTTCTTGT CGCCGCTACA
	GGGCGCGTAC TCCTCTTTTGAGGAACAAGTTTTCTTGT TATGGTTGCTTTGACGAG
Staple 28-31 Substitutes	
	TTGCAACAGG TCCTCTTTTGAGGAACAAGTTTTCTTGT AAAAACGCTC
	ATGGAAATAC TCCTCTTTTGAGGAACAAGTTTTCTTGT CTACATTTTG
	ACGCTCAATC TCCTCTTTTGAGGAACAAGTTTTCTTGT GTCTGAAATG
	GATTATTTAC TCCTCTTTTGAGGAACAAGTTTTCTTGT ATTGGCAGAT
	TCACCAGTCA TCCTCTTTTGAGGAACAAGTTTTCTTGT CACGACCAGT
	AATAAAAGGG TCCTCTTTTGAGGAACAAGTTTTCTTGT ACATTCTGGC
	CAACAGAGATAGAACCCTTCTGACCTGAAAGC
Staple 40-43 Substitutes	
	CACTAACAAC TCCTCTTTTGAGGAACAAGTTTTCTTGT TAATAGATTA
	GAGCCGTCAA TCCTCTTTTGAGGAACAAGTTTTCTTGT TAGATAATAC
	ATTTGAGGAT TCCTCTTTTGAGGAACAAGTTTTCTTGT TTAGAAGTAT
	TAGACTTTAC TCCTCTTTTGAGGAACAAGTTTTCTTGT AAACAATTCG
	ACAACTCGTA TCCTCTTTTGAGGAACAAGTTTTCTTGT TTAAATCCTT
	TGCCCGAACG TCCTCTTTTGAGGAACAAGTTTTCTTGT TTATTAATTT
	TAAAAGTTTG AGTAACATTA TCATTTTGCGGA
Staple 52-55 Substitutes	
	TTACCTGAGC TCCTCTTTTGAGGAACAAGTTTTCTTGT AAAAGAAGAT
	GATGAAACAA TCCTCTTTTGAGGAACAAGTTTTCTTGT ACATCAAGAA
	AACAAAATTA TCCTCTTTTGAGGAACAAGTTTTCTTGT ATTACATTTA
	ACAATTTCAT TCCTCTTTTGAGGAACAAGTTTTCTTGT TTGAATTACC
	TTTTTTAATG TCCTCTTTTGAGGAACAAGTTTTCTTGT GAAACAGTAC
	ATAAATCAAT TCCTCTTTTGAGGAACAAGTTTTCTTGT ATATGTGAGT

	GAATAACCTTGCTTCTGTAAATCGTCGCTATT
Staple 64-69 Substitutes	
	TTACTAGAAA TCCTCTTTTGAGGAACAAGTTTTCTTGT AAGCCTGTTT
	AGTATCATAT TCCTCTTTTGAGGAACAAGTTTTCTTGT GCGTTATACA
	AATTCTTACC TCCTCTTTTGAGGAACAAGTTTTCTTGT AGTATAAAGC
	CAACGCTCAA TCCTCTTTTGAGGAACAAGTTTTCTTGT CAGTAGGGCT
	TAATTGAGAA TCCTCTTTTGAGGAACAAGTTTTCTTGT TCGCCATATT
	TAACAACGCC TCCTCTTTTGAGGAACAAGTTTTCTTGT AACATGTAAT
	TTAGGCAGAG TCCTCTTTTGAGGAACAAGTTTTCTTGT GCATTTTCGA
	GCCAGTAATA TCCTCTTTTGAGGAACAAGTTTTCTTGT AGAGAATATA
	AAGTACCGAC TCCTCTTTTGAGGAACAAGTTTTCTTGT AAAAGGTAAA
	GTAATTCTGT TCCTCTTTTGAGGAACAAGTTTTCTTGT CCAGACGACG
	ACAATAAACA TCCTCTTTTGAGGAACAAGTTTTCTTGT ACATGTTCAGCTAATGCA

[a]Sequence of 190 staplers can be found in previous work.[1]

Table S3. Sequences of dumbbells used at sensing area.[a]

Staple 96-99 Substitutes for sensing site A1/W1	
	CTTGAGCCAT TCCTCTTTTGAGGAACAAGTTTTCTTGT TTGGGAATTA
	GAGCCAGCAA TCCTCTTTTGAGGAACAAGTTTTCTTGT AATCACCAGT
	AGCACCATTA TCCTCTTTTGAGGAACAAGTTTTCTTGT CCATTAGCAA
	GGCCGGAAAC TCCTCTTTTGAGGAACAAGTTTTCTTGT GTCACCAATG
	AAACCATCGA TCCTCTTTTGAGGAACAAGTTTTCTTGT TAGCAGCACC
Probe A1	GTAATCAGTAGCGAC AGAAT XXXXXXXXXXXXXXXXXXXX
	CAAGTTTGCCTTTAGCGTCAGACTGTAGCGCG
Staple 103-106 Substitutes for sensing site A2/W2	
	TCAGAACCGCCACCCTCAGAGCCACCACCCTC
Probe A2	XXXXXXXXXXXXXXXXXXXX AGAGC CGCCACCAGAACCAC
	CACCAGAGCC TCCTCTTTTGAGGAACAAGTTTTCTTGT GCCGCCAGCA
	TTGACAGGAG TCCTCTTTTGAGGAACAAGTTTTCTTGT GTTGAGGCAG
	GTCAGACGAT TCCTCTTTTGAGGAACAAGTTTTCTTGT TGGCCTTGAT
	ATTCACAAAC TCCTCTTTTGAGGAACAAGTTTTCTTGT AAATAAATCC
	TCATTAAAGC TCCTCTTTTGAGGAACAAGTTTTCTTGT CAGAATGGAA
Staple 119-122 Substitutes for sensing site B1/X1	
	CAGACAGCCC TCCTCTTTTGAGGAACAAGTTTTCTTGT TCATAGTTAG
	CGTAACGATC TCCTCTTTTGAGGAACAAGTTTTCTTGT TAAAGTTTTG
	TCGTCTTTCC TCCTCTTTTGAGGAACAAGTTTTCTTGT AGACGTTAGT
	AAATGAATTT TCCTCTTTTGAGGAACAAGTTTTCTTGT TCTGTATGGG
	ATTTTGCTAA TCCTCTTTTGAGGAACAAGTTTTCTTGT ACAACTTTCA
Probe B1	ACAGTTTCAGCGGAG TGAGA XXXXXXXXXXXXXXXXXXXX
	ATAGAAAGGAACAACTAAAGGAATTGCGAAT
Staple 126-129 Substitutes for sensing site B2/X2	
	GTTGCGCCGACAATGACAACAACCATCGCCCA
Probe B2	XXXXXXXXXXXXXXXXXXXX CGCAT AACCGATATATTCGG
	TCGCTGAGGC TCCTCTTTTGAGGAACAAGTTTTCTTGT TTGCAGGGAG

	TTAAAGGCCG TCCTCTTTTGAGGAACAAGTTTTCTTGT CTTTTGCGGG
	ATCGTCACCC TCCTCTTTTGAGGAACAAGTTTTCTTGT TCAGCAGCGA
	AAGACAGCAT TCCTCTTTTGAGGAACAAGTTTTCTTGT CGGAACGAGG
	GTAGCAACGG TCCTCTTTTGAGGAACAAGTTTTCTTGT CTACAGAGGC
Staple 142-145 Substitutes for sensing site C1/Y1	
	GATGGTTTAA TCCTCTTTTGAGGAACAAGTTTTCTTGT TTTCAACTTT
	AATCATTGTG TCCTCTTTTGAGGAACAAGTTTTCTTGT AATTACCTTA
	TGCGATTTTA TCCTCTTTTGAGGAACAAGTTTTCTTGT AGAACTGGCT
	CATTATACCA TCCTCTTTTGAGGAACAAGTTTTCTTGT GTCAGGACGT
	TGGGAAGAAA TCCTCTTTTGAGGAACAAGTTTTCTTGT AATCTACGTT
Probe C1	AATAAAACGAACTAA CGGAA XXXXXXXXXXXXXXXXXXXX
	CAACATTATTACAGGTAGAAAGATTCATCAGT
Staple 149-152 Substitutes for sensing site C2/Y2	
	CCAAAATAGCGAGAGGCTTTTGCAAAAGAAGT
Probe C2	XXXXXXXXXXXXXXXXXXXX TTTGC CAGAGGGGGTAATAG
	TAAAATGTTT TCCTCTTTTGAGGAACAAGTTTTCTTGT AGACTGGATA
	GCGTCCAATA TCCTCTTTTGAGGAACAAGTTTTCTTGT CTGCGGAATC
	GTCATAAATA TCCTCTTTTGAGGAACAAGTTTTCTTGT TTCATTGAAT
	CCCCCTCAAA TCCTCTTTTGAGGAACAAGTTTTCTTGT TGCTTTAAAC
	AGTTCAGAAA TCCTCTTTTGAGGAACAAGTTTTCTTGT ACGAGAATGA
Staple 165-168 Substitutes for sensing site D1/Z1	
	CAATAAATCA TCCTCTTTTGAGGAACAAGTTTTCTTGT TACAGGCAAG
	GCAAAGAATT TCCTCTTTTGAGGAACAAGTTTTCTTGT AGCAAAATTA
	AGCAATAAAG TCCTCTTTTGAGGAACAAGTTTTCTTGT CCTCAGAGCA
	TAAAGCTAAA TCCTCTTTTGAGGAACAAGTTTTCTTGT TCGGTTGTAC
	CAAAAACATT TCCTCTTTTGAGGAACAAGTTTTCTTGT ATGACCCTGT
Probe D1	AATACTTTTGCGGGA GAAGC XXXXXXXXXXXXXXXXXXXX
	CTTTATTTCAACGCAAGGATAAAAATTTTTAG
Staple 172-175 Substitutes for sensing site D2/Z2	
	CTGATAAATTAATGCCGGAGAGGGTAGCTATT
Probe D2	XXXXXXXXXXXXXXXXXXXX TTTGA GAGATCTACAAAGGC
	TATCAGGTCA TCCTCTTTTGAGGAACAAGTTTTCTTGT TTGCCTGAGA
	GTCTGGAGCA TCCTCTTTTGAGGAACAAGTTTTCTTGT AACAAGAGAA
	TCGATGAACG TCCTCTTTTGAGGAACAAGTTTTCTTGT GTAATCGTAA
	AACTAGCATG TCCTCTTTTGAGGAACAAGTTTTCTTGT TCAATCATAT
	GTACCCCGGT TCCTCTTTTGAGGAACAAGTTTTCTTGT TGATAATCAG

[a] Sensing overhangs in the probes are changed based on the target sequences as shown in Table S1.

Table S4. Probes used at different sensing sites (A, B, C, and D) for different DNA carriers.

Carriers	Site A1/W1	Site A2/W2	Site B1/X1	Site B2/X2	Site C1/Y1	Site C2/Y2	Site D1/Z1	Site D2/Z2
Carrier in Figure 1	P1W	P2W	None	None	None	None	None	None
Carrier in Figure 2	P1W	P2W	P1X	P2X	P1Y	P2Y	P1Z	P2Z
Carrier 000 in	PA1G	PA2F	None	None	None	None	None	None

Figure 3								
Carrier 001 in Figure 3	PA1T	PA2F	None	None	None	None	None	None
Carrier 010 in Figure 3	PA1A	PA2F	None	None	None	None	None	None
Carrier 011 in Figure 3	PA1C	PA2F	None	None	None	None	None	None
Carrier 1 in Figure 4	None	None	PB1a1	PB2a1	PC1b1	PC2b1	None	None
Carrier 2 in Figure 4	None	None	PB1a2	PB2a2	PC1b2	PC2b2	None	None
Carrier 3 in Figure 4	None	None	PB1a3	PB2a3	PC1b3	PC2b3	None	None
Carrier for small loop in Figure S3 and S7	PA1M	PA2M	None	None	None	None	None	None
Carrier for large loop in Figure S3	PA1M	None	None	PB2M	None	None	None	None

Table S5. Statistics of translocation events of carrier in Figure 1 and S5.

Sample	I_0 (nA)	Total number of analyzed events	Number of events with single peak[a]	Single peak fraction (SPF)
Carrier only (Blank)	0.119	151	8	0.0530
	0.193	138	5	0.0362
	0.173	135	6	0.0444
Carrier + 2.5 nM T1	0.125	150	94	0.627
	0.179	156	104	0.667
	0.176	157	116	0.739
Carrier + 2.5 nM T2	0.144	150	14	0.0933
	0.139	142	11	0.0775
	0.160	154	9	0.0584
Carrier + 2.5 nM T3	0.162	138	18	0.130
	0.143	152	12	0.0789
	0.146	164	22	0.134
Carrier + 2.5 nM T4	0.145	103	22	0.223
Carrier + 2.5 nM T5	0.171	177	38	0.215
Carrier + 2.5 nM T6	0.153	101	55	0.545
Carrier + 0.25 nM T1	0.182	179	30	0.168
	0.134	149	24	0.161
	0.131	150	30	0.200
Carrier + 0.625 nM T1	0.109	150	70	0.467
	0.153	157	55	0.350

	0.177	147	85	0.578
Carrier + 1.25 nM T1	0.154	147	93	0.633
	0.133	174	89	0.511
	0.219	107	52	0.486
Carrier + 6.25 nM T1	0.169	129	84	0.651
	0.162	160	116	0.725
	0.198	86	67	0.779

Table S6. Statistics of translocation events of carrier in Figure 2.

Sample name	I_0 (nA)	Total number of analyzed events	Number of events with single peak at site W	Number of events with single peak at site X	Number of events with single peak at site Y	Number of events with single peak at site Z
Only carrier (Blank)	0.148	139	4	0	1	2
	0.109	77	1	0	1	1
	0.166	104	1	1	1	1
Carrier + target W + Y	0.102	128	55	1	45	0
	0.144	130	56	0	35	2
	0.190	93	19	2	46	1
Carrier + target X + Z	0.102	104	0	23	1	46
	0.133	130	2	14	2	76
	0.173	101	2	12	1	53
Carrier + four targets	0.124	120	48	18	32	45
	0.168	130	64	15	41	65
	0.183	72	23	11	17	32

Table S7. Statistics of translocation events of carrier in Figure 3.

Sample name	I_0 (nA)	SPF of carrier 000	SPF of carrier 001	SPF of carrier 010	SPF of carrier 011
Carrier mixture + dG	0.157	25/39	3/38	9/51	3/73
	0.151	24/52	4/38	4/33	5/62
	0.155	59/103	4/75	10/90	15/135
Carrier mixture + dT	0.154	16/63	44/59	15/56	29/76
	0.167	16/61	41/66	4/48	14/71
	0.146	24/99	38/51	10/69	34/92
Carrier mixture + dA	0.143	2/22	7/94	55/80	36/89
	0.142	7/62	7/66	64/80	60/111
	0.139	10/56	11/50	41/63	37/77
Carrier mixture + dC	0.121	2/40	6/37	6/41	44/67
	0.143	6/43	4/47	6/44	63/88
	0.154	4/47	2/36	11/39	63/79

Carrier mixture + rG	0.144	19/58	1/59	3/56	1/107
	0.202	7/22	1/22	1/18	2/40
	0.150	7/33	1/28	0/23	0/42

Table S8. Statistics of translocation events of carrier in Figure 4.

Sample name	I_0 (nA)	Carrier 000		Carrier 010		Carrier 011	
		SPF at Site B	SPF at Site C	SPF at Site B	SPF at Site C	SPF at Site B	SPF at Site C
Carrier mixture + *E. coli* DH5α RNA fragments	0.147	4/64	7/64	1/78	3/78	0/50	1/50
	0.164	7/80	12/80	1/105	7/105	5/66	1/66
	0.172	10/69	5/69	3/77	3/77	1/62	2/62
Carrier mixture + *Salmonella* RNA fragments	0.141	0/64	1/64	6/64	9/64	2/82	1/82
	0.144	1/34	1/34	4/48	5/48	1/46	1/46
	0.145	2/40	1/40	7/45	3/45	0/37	1/37
Carrier mixture + *Acinetobacter baumannii* RNA fragments	0.174	0/64	1/64	1/46	1/46	0/75	3/75
	0.105	2/44	1/44	2/59	1/59	1/56	2/56
	0.160	1/44	0/44	0/43	1/43	0/35	1/35

Table S9. Statistics of translocation events of carrier in Figure S3 and S7.

Sample	I_0 (nA)	Total number of analyzed events	Number of events with single peak[a]	Single peak fraction (SPF)
Carrier only (Blank)	0.104	103	3	0.029
Carrier + 2.5 nM M	0.145	112	57	0.509
Carrier (large loop) + 2.5 nM M	0.113	122	32	0.262
Carrier + 1.47 nM MS2 RNA	0.125	126	6	0.0476
Carrier + 4.41 nM MS2 RNA	0.143	89	7	0.0787
Carrier + 7.35 nM MS2 RNA	0.152	113	11	0.0973
Carrier + 14.7 nM MS2 RNA	0.125	105	10	0.0952
Carrier + 23.5 nM MS2 RNA	0.140	87	10	0.115

Reference

[1] Bell, N. A. W.; Keyser, U. F. Digitally encoded DNA nanostructures for multiplexed, single-molecule protein sensing with nanopores. *Nat. Nanotechnol.* **2016,** *11*, 645.

[2] Zhu, J.; Ermann, N.; Chen, K.; Keyser, U. F. Image encoding using multi-level DNA barcodes with nanopore readout. *Small* **2021,** *17*, 2100711.
